# Adherence, Competence, and Alliance as Predictors of Long-term Outcomes of Cognitive Behavioral Therapy for Youth Anxiety Disorders

**DOI:** 10.1007/s10802-023-01028-1

**Published:** 2023-01-24

**Authors:** Jon Fauskanger Bjaastad, Rolf Gjestad, Krister Fjermestad, Lars-Göran Öst, Bente Storm Mowatt Haugland, Arne Kodal, Einar R. Heiervang, Gro Janne Wergeland

**Affiliations:** 1 Division of Psychiatry, Stavanger University Hospital, N-4068 Stavanger, Norway; 2 Research Department, Division of Psychiatry, Haukeland University Hospital, N-5021 Bergen, Norway; 3 Centre for Research and Education in Forensic Psychiatry, Haukeland University Hospital, N-5021 Bergen, Norway; 4 Centre for Crisis Psychology, Faculty of Psychology, University of Bergen, N-5021 Bergen, Norway; 5 Department of Psychology, University of Oslo, N-0317 Oslo, Norway; 6 Department of Psychology, Stockholm University, SE-106 91 Stockholm, Sweden; 7 Department of Clinical Psychology, Faculty of Psychology, University of Bergen, N-5020 Bergen, Norway; 8 Department of Child and Adolescent Psychiatry, Division of Psychiatry, Haukeland University Hospital, N-5021 Bergen, Norway; 9 Institute of Clinical Medicine, Faculty of Medicine, University of Oslo, N-0315 Oslo, Norway; 10 Department of Mental Health & Addiction, Oslo University Hospital, N-0424 Oslo, Norway; 11 Department of Clinical Medicine, Faculty of Medicine, University of Bergen, N-5020 Bergen, Norway

**Keywords:** Alliance, Adherence, Competence, Cognitive behavior therapy, Youth, Anxiety

## Abstract

The present study investigated therapist adherence, therapist competence, and patient-therapist alliance as predictors of long-term outcomes of cognitive behavioral therapy (CBT) for anxiety disorders in youth. Potential differential effects for group versus individual CBT, for therapists with or without formal CBT training, and based on youth symptom severity were examined. Videotapes (*n* = 181) from treatment sessions in a randomized controlled effectiveness trial comprising youth (*N* = 170, *M age =* 11.6 years, *SD* = 2.1) with anxiety disorders were assessed for therapist adherence and competence. Alliance was rated by therapists and youth. Participants completed a diagnostic interview and an anxiety symptom measure at pre-treatment, post-treatment, one-year follow-up, and long-term follow-up (*M* = 3.9 years post-treatment, *SD* = 0.8, range = 2.2–5.9 years). The change in anxiety symptoms or diagnostic status from pre-treatment to long-term follow-up was not significantly related to any predictor variables. However, several interaction effects were found. For loss of principal diagnosis, therapist competence predicted positive outcome when therapist adherence also was high. Adherence was found to predict positive outcome if CBT was provided individually. Therapist-rated alliance was related to both loss of principal diagnosis and loss of all diagnoses when CBT was provided in groups. Interaction effects suggested that therapists displaying both high adherence and high competence produced better long-term outcomes. Further, the alliance may be particularly important for outcomes in group CBT, whereas adherence may be particularly important for outcomes in individual CBT.

**Supplementary Information:**

The online version contains supplementary material available at 10.1007/s10802-023-01028-1.

## Adherence, Competence and Alliance As Predictors of Long-Term Outcome in CBT for Anxiety Disorders in Youth

Anxiety disorders in youth are highly prevalent and associated with significant functional impairment (Costello et al., 2003; Merikangas et al., 2010; Swan & Kendall, 2016). Cognitive behavioral therapy (CBT) is a well-established treatment for anxiety disorders in youth, with favorable outcomes across treatment modalities and settings including specialized clinics (James et al., 2020) and routine clinical care (Wergeland et al., 2021). However, a large proportion of youth are non-responders at post-treatment, or not in stable remission at long-term follow up (Ginsburg et al., 2018; Kodal et al., 2018a). This indicates a need for more effective interventions. One way to potentially improve CBT is to investigate therapist effects (e.g., therapist adherence to the manual and therapist competence in delivering the intervention) to identify potential targets for training and supervision of therapists (Baldwin & Imel, 2013). Therapist adherence and competence have been suggested as candidates for quality indicators of treatment implementation in CBT for youth anxiety, which could potentially improve treatment service (McLeod et al., 2013). Quality indicators could be process variables in treatment (e.g., therapist adherence, competence, and alliance) that have demonstrated improvement in patient outcomes (McLeod et al., 2013). These process variables could be measured, monitored, and targeted (e.g., increase training if competence levels drop below a certain threshold) to provide better services. However, although therapist adherence and competence may potentially be related to long-term follow up outcomes, no studies to date have investigated this for CBT for anxiety disorders in youth.

Therapist adherence is defined as the degree to which the therapist applies prescribed procedures and avoids non-prescribed procedures, whereas therapist competence is defined as the level of the therapist’s skills in delivering the treatment interventions (Perepletchikova et al., 2007). To date, three meta-analyses have investigated the role of therapist adherence and competence as predictors of outcome. The first did not find support for therapist adherence and competence as predictors of outcome for adult patients, with results yielding small and non-significant effect sizes (Webb et al., 2010). However, none of the competence-outcome studies and only three of the 32 adherence-outcome studies in this meta-analysis were based on CBT studies, and none of the included studies were primarily targeting anxiety disorders. The second meta-analysis (Zarafonitis-Müller et al., 2014) included 13 CBT studies on adult and adolescent samples and therapist competence was found to have a small, but significant effect on outcomes (*r* = .24), whereas adherence had no effect on outcomes. The third meta-analysis included 35 studies of psychotherapy for children and adolescents (Collyer et al., 2019). A small but significant association (*r* = .096), between adherence and outcome was found, but no significant association was found between competence and outcome. However, only seven of the 35 included studies were CBT studies and only three studies targeted anxiety disorders (Collyer et al., 2019).

In CBT for youth anxiety, the avoidance often exhibited by patients, may require particular therapist skills, and hence give therapist’s adherence and competence a particularly important role. For instance, since conducting exposures is considered a main intervention in CBT for anxiety disorders, adherence and competence related to conducting exposures may be particularly relevant, as avoidance is linked to anxiety, and exposures are often anxiety-provoking and may require specific therapist skills. Youth may be less motivated to do exposure tasks than adults as treatment is often initiated by their parents rather than based on the youth’s own wish. This may put extra demand on therapist skills in motivating, using developmentally appropriate language to explain the rationale behind exposure, and helping the youth with conducting exposures. However, there is limited research investigating the relationship between therapist adherence and competence on outcomes for CBT for anxiety disorders in youth, and findings are mixed (Bjaastad et al., 2018; Hudson et al., 2014; Liber et al., 2010; Podell et al., 2013; Southam-Gerow et al., 2021). All studies to date are mainly on samples of White youth and therapists which may limit generalization to more diverse populations. Only two studies have used adherence and competence scales with reported psychometric properties. The first study found that higher therapist adherence was related to better treatment outcomes in youth aged 8–15, whereas competence was related to poorer post-treatment outcomes (Bjaastad et al., 2018). However, this study reported interaction effects suggesting that competence in therapists with formal CBT training was related to better outcomes. This suggests the need to examine interaction effects when evaluating the role of adherence and competence in predicting outcome. The second study found no statistically significant relationship between neither adherence nor competence and post-treatment outcome in youth aged 7–14 (Southam-Gerow et al., 2021). As there are few studies conducted and results so far are inconsistent (Collyer et al., 2019; Rapley & Loades, 2018), more studies are needed regarding the role of adherence and competence on outcome for youth anxiety disorders in general, and particularly for treatment in routine clinical care. Importantly, there are to date no studies investigating therapist adherence and competence as predictors of long-term outcomes.

One potential reason that the studies on adherence and competence provide such mixed findings is that variables likely to interact with these therapist effects in affecting outcomes are not included. Several variables are likely to influence how adherence and competence affect outcomes. The current study included the alliance, defined as a collaborative bond between therapist and youth receiving treatment (Karver et al., 2018). Studies indicate that the effect sizes for treatment adherence and competence on outcome are smaller when the alliance is controlled for (Webb et al., 2010). The inclusion of the alliance as an additional predictor in studies investigating the role of adherence and competence on outcome in youth has therefore been recommended (Collyer et al., 2019). In child and adolescent psychotherapy, meta-analyses have found alliance to be associated with small to medium effects on outcomes (mean weighted *r* = .14 in McLeod 2011; and weighted random effect size of *r* = .19 in Karver et al., 2018).

In addition to including the alliance as a predictor, the current study also considered several other treatment factors that may influence the interplay between adherence, competence, and alliance for outcomes. The first is treatment format. Treatment format (individual or group CBT) has been found to provide comparable effects regarding youth anxiety treatment (James et al., 2015). However, it may be that adherence, competence and alliance are associated with treatment format in different ways, e.g., that alliance may play a different role in group CBT where the therapist may have less interaction with each youth than in individual therapy. Thus, we need to investigate potential interaction effects between treatment format and adherence, competence and alliance. Developmental issues and type of problem may also affect the association of treatment format with adherence, competence and alliance. For example, an adolescent who is inactive in CBT due to high level of social anxiety, versus a child who finds it hard to follow treatment due to developmental differences in attention, could place different demands on therapist skills (e.g., developmental sensitivity, flexibility) pending on whether the youth received group CBT or individual CBT. Adolescents may also be more active in groups than younger children due to their increased peer-orientation, and the increased need for autonomy during adolescence needs to be planned for across treatment formats. Due to growing needs for autonomy during adolescence it may also be challenging to engage adolescents in CBT tasks and parental involvement in both group CBT and individual CBT.

The second factor we included was baseline symptom severity. Higher symptom-severity at pre-treatment has been found to be a predictor of poorer CBT outcomes in youth with anxiety and depressive disorders (Kunas et al., 2021). High levels of symptom-severity may put more demand on the therapist regarding completion of session tasks, such as exposure exercises. Thus, symptom severity should be included when investigating predictors of outcome in CBT for youth anxiety.

Finally, we also considered therapist formal CBT training, which has been suggested to be related to better outcomes (Rakovshik & McManus, 2010). In CBT for youth anxiety disorders, one study found that levels of therapist CBT training, defined as “novice” (no previous experience of using CBT or clinical experience) or “some clinical training” (currently enrolled in clinical training or previous clinical experience) was unrelated to outcome in guided parent-delivered CBT for child (7–12 years) anxiety disorders (Thirlwall et al., 2013). Another study on mixed anxiety disorders in youth (8–15 years old) found that formal CBT training among therapists (defined as a two-year part-time CBT training) was associated with greater youth-reported symptom change at post-treatment (Bjaastad et al., 2018). This shows mixed findings also for CBT training. A study on CBT for adults with anxiety disorders found an interaction effect between symptom severity and level of therapist CBT training, indicating that for more severely anxious patients, qualified therapists (9 clinical psychologists and 5 CBT therapists, *n* = 14) achieved greater anxiety symptom reduction than trainee therapists (therapists under training for these professions, *n* = 20) (Mason et al., 2016). This finding highlights the importance of investigating interaction effects, and the need to include predictors such as symptom severity and formal CBT training.

To sum up, there is limited knowledge regarding the effect of adherence, competence and alliance on treatment outcome in CBT for youth anxiety, interaction effects may be present, and studies on long-term follow-up are lacking. Most studies to date have also been carried out in university clinic settings and not in routine clinical care, and there may be differences between these settings in patient characteristics (e.g., it has been found that patients in routine clinical care have more severe symptoms and are more functionally impaired) (Villabø et al., 2013) and therapist characteristics (e.g., assumption of CBT-trained clinicians with focused caseloads in university clinic settings). Therefore, more research on predictors of long-term outcome of youth with anxiety in routine clinical care are warranted.

The aim of the present study was to investigate whether therapist adherence, therapist competence, and patient-therapist alliance predict long-term treatment outcome in CBT for youth with anxiety disorders in community clinics. Given that prior research suggests interaction effects between these variables and treatment format, baseline symptom severity and formal CBT training of therapists, we also investigate these effects.

The current study is a long-term follow-up study (*M* = 3.9 years post-treatment) that extends on a prior effectiveness study examining therapist adherence, competence, formal CBT training and clinical experience on outcome in CBT for youth anxiety (Bjaastad et al., 2018). We included therapist adherence, therapist competence and alliance as predictor variables of long-term outcome and also investigated interaction effects. We examined the following research questions: (1) Do therapist adherence, therapist competence, and patient-therapist alliance predict outcomes at long-term follow-up? (2) Will therapist adherence, therapist competence, and patient-therapist alliance predict long-term outcome differentially based on treatment format, baseline symptom severity and therapists’ formal CBT training? Due to limited studies of adherence and competence as predictors in CBT for anxiety disorders in youth, and mixed results across the few previous studies, the research questions relating to adherence and competence were explored openly. However, based on earlier research on the well-established alliance-outcome association, we expected the alliance to be related to long-term outcomes. Due to lack of research investigating interaction effects of treatment format, baseline symptom severity and therapists’ formal CBT training on long-term outcome, these research questions were also explored openly.

## Methods

This study is part of a randomized controlled effectiveness trial (RCT) of individual CBT (ICBT) and group CBT (GCBT) for anxiety disorders in youth. Details on methods and main outcomes have been described elsewhere and will not be presented in detail (Kodal et al., 2018a; Wergeland et al., 2014). The study was approved by the Regional Committees for Medical and Health Research Ethics, Western Norway and registered in clinicaltrials.gov (NCT00586586).

### Participants and Procedures

A total of 182 youth (8–15 years old) were initially recruited from referrals to seven public youth mental health outpatient clinics, including both urban and rural community sites. Inclusion criteria was having a principal disorder of separation (SAD)-, social (SOP)-, or generalized anxiety disorder (GAD) according to the DSM-IV (American Psychiatric Association, 1994). Exclusion criteria were the presence of pervasive developmental disorder, known learning disability or psychotic disorder. Groups of six youth included at a clinic, either from the younger age group (8–12 years) or from the older age group (12–15 years) were randomly assigned to ICBT, GCBT, or to a waitlist control group. The mean age was 11.6 years (*SD =* 2.1) for youth participating in the study (47.1% male, 52.9% female). Participants identified as mainly White (90.7%), Asian (1.6%), and for 7.7% race was not reported.

Participants who met criteria for inclusion after waitlist were randomized to ICBT or GCBT. Written consent from all parents and assent from youth from ages 12–15 years was secured.

In the RCT, two participants dropped out while on waitlist and one participant was diagnosis-free after waitlist, resulting in a final sample of 179 youth of whom 88 were randomized to GCBT and 91 to ICBT (Wergeland et al., 2014). In the current sample video recordings were available for 170 youth. See Table 1 for baseline characteristics for these participants.

**Table 1:** Participants Characteristics at Baseline (N = 170)

		N	(%)	M	(SD)
Age				11.6	(2.1)
Female		90	(52.9)		
Single parent status		35	(20.5)		
Family social class*					
	High	53	(31.2)		
	Medium	87	(51.2)		
	Low	14	(8.2)		
Principal anxiety disorder					
	SAD	57	(33.5)		
	SOP	78	(45.9)		
	GAD	35	(20.6)		
Comorbid anxiety**		119	(70.0)		
Non-anxiety comorbidity		61	(36.3)		
SCAS-C				36.4	(16.7)
SCAS-P				35.0	(12.5)

*Note.* Observed proportions, means, and standard deviation of demographic characteristics, and for youth- and parent reported diagnostic and symptom measures. SAD = Separation anxiety disorder; SOP = Social anxiety disorder; GAD = Generalized anxiety disorder; SCAS = Spence Child Anxiety Scale; C = child; P = parent; *Registrar General Social Class coding scheme, Currie et al., (2008) **Proportion of youth with ≥ 1 inclusion anxiety disorder.

Youth and parents completed assessments at pre-treatment (T1), post-treatment (T2), one-year follow-up (T3), and long-term follow-up (T4) (mean = 3.9 years post-treatment, range = 2.2–5.9 years). Youth who participated in the long-term follow-up assessment were between 11 and 21 years (*M* = 15.5, *SD* = 2.5, 54.7% female) and comprised 77.7% of the total initial sample, with 90.3% being treatment completers (Kodal et al.,2018a). Participants at long-term follow-up did not differ significantly from youth who did not partake in the long-term follow-up assessment (*n* = 40) in terms of pre-treatment socio-demographic characteristics, pre-treatment self-reported (parent and youth reported) clinical variables, and presence of principal inclusion anxiety disorder at pre-treatment (Kodal et al.,2018a).

### Treatment

The “Friends for life” (Barrett, 2004) program was used in this study. This is a 10-session manual-based CBT program for youth addressing cognitive, physiological, and behavioral components that interact in the development and maintenance of anxiety. The program is developed for use both as ICBT and GCBT (Barrett, 2004). Parents attended two separate parent sessions, two of the ten sessions for youth, as well as the last 15 min of the remaining eight sessions.

### Therapists, Diagnostic Assessors and Raters of Adherence and Competence

Seventeen therapists (*M* age = 48.2 years, *SD* = 11.0, *range* 30–63, 94.0% female) participated. All therapists (ten psychologists, six special educators with clinical training and one social worker) were regular clinicians working at the community clinics. They conducted the treatment as part of their ordinary workload. Each therapist treated a median of 10 youth (*range* 2–17). Seven therapists had completed a part-time formal CBT training over two years, whereas the other therapists had little or no formal CBT training prior to the study. All therapists attended a two-day workshop about CBT and youth anxiety disorders and a two-day training in the treatment manual. Each therapist delivered two pilot treatments, that were approved by the supervisors prior to commencement of the study. During the study, therapists participated in four additional two-day workshops on topics related to anxiety disorders in youth. They received regular supervision by one of two experienced CBT therapists, both licensed “Friends for life” trainers. Supervision was given every 2–4 weeks, with an average of 78.7 (*SD* = 34.0) supervision hours per therapist during the study. All therapists administered both ICBT and GCBT, with two therapists assigned to each group of GCBT.

Diagnostic assessors (*n* = 16) were experienced clinicians employed at the participating clinics. They received training in the Anxiety Disorder Interview Schedule for DSM-IV, child and parent versions (ADIS-C/P; Silverman & Albano 1996) during a two-day workshop with licensed ADIS-C/P raters and were supervised on the administration and scoring of the ADIS-C/P throughout the study. At long-term follow-up, diagnostic assessors were three certified interviewers (two psychologists and one child psychiatrist) who were masked to the youths’ inclusion anxiety diagnosis and treatment format, as well as treatment outcome both at post-treatment and 1-year follow-up.

Raters (*n* = 4) of therapist adherence and competence were two experienced CBT therapists and two graduate psychology students who were trained in the “Friends for life” program. The two CBT therapists developed the Competence and Adherence Scale for Cognitive Behavioral Therapy (CAS-CBT; Bjaastad et al., 2016) used for rating adherence and competence. The students received training in the CAS-CBT from its’ developers. The student raters were tested for accuracy before being included as raters. Two of five recruited student raters achieved satisfactory accuracy according to Cicchetti (1994), with expert ratings on 10 videos used for accuracy testing (ICCs = 0.90 and 0.75 for adherence indicating excellent agreement, and ICCs = 0.50 and 0.41 for competence indicating fair agreement). These two were used as student raters in the study. None of the raters worked in any of the clinics where the treatment was provided. All therapy sessions were videotaped and 20% were randomly selected for adherence and competence ratings. A total of 181 videos were scored, of which 127 videos were scored by the student scorers (student 1 = 74, student 2 = 53) and 54 videos by the expert scorers. The videos were randomly assigned to the raters. Selection of sessions was stratified on early (session 2–5) and late (session 6–9) sessions, with each therapist being represented with at least three sessions (*M =* 10.7, *range* 3–18). A total of 26 randomly selected student scored videos (20% of videos scored by students) were randomly allocated to expert scorers for interrater reliability (IRR) evaluation. IRR was excellent (ICC = 0.83) for adherence and good (ICC = 0.64) for competence. See Bjaastad et al., (2018) for more details on the ratings of therapist adherence and competence.

### Measures

**The Competence and Adherence Scale for Cognitive Behavioral Therapy for Anxiety Disorders in Youth** (CAS-CBT; Bjaastad et al., 2016) measures therapist adherence and competence in delivering CBT for youth. The measure assesses CBT structure (agenda setting, homework tasks, progress and structure, parental involvement), process and relational skills (positive reinforcement, cooperation, flexibility), and the two main goals of each session (e.g., core components such as planning exposures, cognitive restructuring, skills training, problems solving) in the “Friends for life” program (Barrett, 2004). Adherence was rated from 0 (“None”) to 6 (“Thorough”), and competence from 0 (“Poor skills”) to 6 (“Excellent skills”). CAS-CBT has demonstrated good to excellent inter-rater reliability, high rater stability and a factor structure comprising two factors (Bjaastad et al., 2016). Satisfactory reliability and replication of the factor structure has also been demonstrated for both GCBT and ICBT for youth anxiety, with another manualized CBT for youth anxiety program (Harstad et al., 2021). The internal consistency for the CAS-CBT in the current study was good (α = 0.87).

**The Therapeutic Alliance Scale for Children – Child and Therapist versions** (TASC-C/T; Shirk & Saiz 1992; Accurso et al., 2015), which is a 12-item self-reported alliance inventory (e.g., *I liked spending time with my therapist*/*I liked spending time with this child*). TASC-C/T was completed by youth and therapists at the end of sessions 3 and 7 to assess youth- and therapist-rated alliance. Items are scored on a 4-point scale ranging from 1 (*not true at all*) to 4 (*very true*). The TASC-C/T have demonstrated good to excellent internal consistency (α = 0.88 to 0.96; Accurso et al., 2015; Creed & Kendall 2005). In the current study, internal consistency for the TASC-C/T, respectively, were acceptable to good (α = 0.77 and 0.85 in session 3, and α = 0.84 and 0.77 in session 7).

**Formal CBT training** was assessed by a questionnaire asking about the educational background of the therapist, and whether they had received any post-graduate CBT training and supervision. This resulted in a dichotomous rating of having formal CBT training or not. The seven therapists who were rated as having formal CBT training had all completed a two-year part-time CBT training accredited by the Norwegian Association of Cognitive Therapy. The training consisted of 30 full-day lectures over a two-year period, CBT supervision over 80 h, case-presentations, as well as a requirement to write a paper on a CBT relevant topic and to pass a written exam in CBT.

**The Anxiety Disorder Interview Schedule for DSM-IV, child and parent versions** (ADIS-C/P; Silverman & Albano 1996); SAD, SOP, and GAD modules were used to assess diagnoses at pre-, post-, one-year follow-up and long-term follow-up assessment, as well as clinician severity ratings (CSR) which were used as a measure of baseline symptom severity. Youth and parents were interviewed separately, and diagnoses and CSR were assigned based on a combined parent and youth report, according to the manual. At long-term follow-up, the ADIS-IV-L (Brown et al., 1994) was used as a diagnostic interview to assess DSM-IV criteria for SAD, SOP and GAD in youth aged 18 or older (*n* = 32). Adequate interrater reliability was demonstrated (Kodal et al.,2018a; Wergeland et al., 2014).

**The Spence Children’s Anxiety Scale** (Spence, 1998), child and parent versions (SCAS-C, SCAS-P), were administered to assess youth anxiety symptoms at all assessment points. The SCAS comprises 38 items, rated on a four-point scale (0 = never, 3 = always). Validity, internal consistency, and adequate test-retest reliability has previously been demonstrated (Spence, 1998; Spence et al., 2003). Internal consistency for the SCAS in the total sample was good to excellent (α: youth = 0.91, parent = 0.85 pre-treatment and α: youth = 0.92, parent = 0.89 at long-term follow-up).

### Data Analysis

Univariate and bivariate statistics were estimated with IBM SPSS version 26.0 (IBM Corp., 2020) and all models were estimated with Mplus version 8.5 (Muthén & Muthén, 2020). Intra class correlations (ICC) were estimated to determine the degree of clustering related to the site and therapist levels in data. The between-site ICC ranged from 0.002 to 0.045, and the between-therapist ICC from 0.001 to 0.047. These results indicate no substantial problems with clustering effects (Guo, 2005). However, the models accounted for clustering effects in the predictor variables, as patients were treated in groups or individually, giving a situation of partial clustering (Baldwin et al., 2011). Analyses were conducted with the MLR estimator under the missing at random (MAR) assumption, accounting for any non-normality in data (Muthén & Muthén, 2011). The outcome variables were found to be somewhat non-normal for some measurement points, with skewness values for the two outcome variables in the range from 0.56 to 1.59. All four measurement points (T1, T2, T3 and T4) were included in the models.

Latent growth curve modeling (LGM) was used separately on youth- and parent-rated anxiety to model treatment response in anxiety symptoms over time at group and individual levels. Three slope factors were used to represent factorial differences over time, between T1-T2, T1-T3, and T1-T4, to use all available information for estimation. Only the estimated results from the change in T1-T4 (pre- to long term follow-up) were reported. The time spacing between observations varied across individuals. Using all available repeated data increases the precision of the estimates. The prediction models defined time as months. To make the models more parsimonious the prediction models assumed random intercept (pre-treatment level) and fixed slopes (change) (Newsom, 2015).

The slopes were regressed on the predictors, both main effects and pre-specified interaction terms, to assess their influence on symptom change. Prediction models of loss of principal and all diagnoses were based on the logistic regression (logit). An odds ratio greater than one predicts higher odds of diagnostic recovery associated with the predictor. Adherence and competence were highly correlated (*r* = .80); therefore, the competence variable was residualized after regressing this variable on adherence and using the residual variance as a new variable. Thus, the adherence and the residualized competence variables were non-related when entered into the prediction models. Due to one result giving a very wide confidence interval, the continuous predictor variables were standardized and the model re-estimated.

The predictor variables for alliance were calculated using the total score of both early and late alliance (sum score of both sessions assessed for alliance). Separate scores for “therapist-rated alliance” and “youth-rated alliance” were computed as these were separate predictors. The predictor variable “adherence” was the total adherence score based on CAS-CBT, whereas the predictor variable “competence” was the residualized score for total competence score on CAS-CBT. The predictor variables “type of treatment format” (ICBT/GCBT) and “formal CBT training” (yes/no) were dichotomous variables, whereas the predictor variable “symptom severity” was computed using a sum score of CSR for the three inclusion diagnoses at pre-treatment. For outcome variables, youth-rated and parent-rated anxiety symptoms were treated as separate outcome variables, as the youth treatment literature recommends a multi-informant perspective (Ogles, 2013).

In the total sample of N = 170, the highest frequency of missing data was observed for SCAS-C (27%) and SCAS-P (30%) at T4. Regarding the analyses of loss of principal and loss of all diagnoses, missing data were present for 27% of the patients at T4. Some missing data were also present in the predictor variables (up to 11%). However, due to the analysis strategy, all cases were used for estimation of these models, N = 124 and N = 170 in the loss of diagnoses and level and change in the SCAS variables, respectively.

## Results

The therapists’ mean score across their treatments ranged from 3.83 to 5.43 (*M* = 4.57, *SD* = 0.94) for adherence and 3.44 to 5.25 (*M* = 4.30, *SD* = 0.91) for competence. Hence, all therapists obtained a mean score above 3.0 on both factors, which was defined as the minimum level for adequate therapist adherence and competence.

Table 2 shows the bivariate correlations among the predictor variables. The youth-rated alliance was positively correlated with competence. Formal CBT training was positively correlated with adherence and competence. Treatment format (group treatment) was negatively correlated with youth-rated alliance with the therapist.

**Table 2:** Product Moment Correlations between Predictors

	TASCtc	TASCct	Adh	Comp	CBT	CSR
Therapist-rated alliance with youth (TASCtc)	-					
Youth-rated alliance with therapist (TASCct)	0.15	-				
Adherence (Adh)	− 0.03	0.11	-			
Competence (Comp)^a^	0.09	0.20*	0.00	-		
Formal CBT training (CBT)	− 0.05	− 0.06	0.38*	0.20*	-	
Clinical Severity Rating (CSR)	0.14	0.08	− 0.07	0.01	− 0.17	-
Group treatment (Group)	− 0.07	− 0.26*	0.04	− 0.07	0.11	0.05

*Note.* Categorical variables are analyzed with Spearman correlations. Listwise deletion. N = 133. ^a^ Residualized variable after accounting for adherence

### Youth Rated Anxiety Symptoms at Long-Term Follow-Up

The mean change in youth rated anxiety symptoms (SCAS) from pre-treatment to long-term follow-up was found to be -0.22 per month (*p* < .001). Some youth reported more reduction, others less, as indicated by the *SD*_*slope*_ 0.27 for T1-T4. The long-term change from pre-treatment to long-term follow-up for youth rated anxiety symptoms was not found to be significantly related to any predictor variables (Table 3).

**Table 3:** Self-Reported Anxiety Symptoms (SCAS) from Pre-Treatment to Long-Term Follow-Up Predicted by Predictor Variable

		*b*	*p*	*95% CI*	
Intercept		-0.18	0.038	-0.34	-0.01
Therapist-rated alliance with youth (TASCtc)		0.02	0.362	-0.03	0.07
Youth-rated alliance with therapist (TASCct)		0.01	0.577	-0.03	0.05
Adherence (Adh)		-0.17	0.574	-0.77	0.42
Competence (Comp)^a^		-0.11	0.491	-0.41	0.20
Formal CBT training (CBT)		0.12	0.066	-0.01	0.25
Clinician Severity Rating (CSR)		-0.01	0.750	-0.03	0.02
Group treatment (Group)		-0.06	0.113	-0.14	0.02
Adh*Group		0.04	0.804	-0.30	0.39
Adh*Comp		-0.01	0.832	-0.06	0.05
Comp*CBT		-0.05	0.204	-0.14	0.03
Comp*Group		0.10	0.307	-0.09	0.28
Comp*CSR		-0.00	0.395	-0.01	0.01
TASCtc*Group		-0.02	0.311	-0.05	0.02
TASCct*Group		-0.01	0.304	-0.04	0.01

*Note.* Self-reported anxiety symptoms (SCAS) from pre-treatment to long-term follow-up predicted by youth-rated alliance, therapist-rated alliance, adherence, competence (residualized), Formal CBT training, clinician severity rating, and the effect of individual treatment compared to group treatment. Hypothesized interaction terms are included

^a^ Residualized variable after accounting for adherence

### Parent Rated Anxiety Symptoms at Long-Term Follow-Up

The mean change in parent rated anxiety symptoms (SCAS) from pre-treatment to long-term follow-up was found to be -0.26 per month (*p* < .001). Some youth reported more reduction, others less, as indicated by the *SD*_*slope*_ 0.24 for T1-T4. The long-term change from pre-treatment to long-term follow-up for parent rated anxiety symptoms was not found to be significantly related to any predictor variables (see supplementary Table S1 for results).

### Loss of all Diagnoses at Long-Term Follow-Up

Loss of all diagnoses was not found to be significantly related to any of the main effect predictors (i.e., therapist adherence, therapist competence, patient-therapist alliance, treatment format, baseline symptom severity and therapists’ formal CBT training) (Table 4). An interaction effect was found, where loss of all diagnoses was related to therapist-rated alliance with youth for GCBT. This indicated that higher levels in therapist-rated alliance with youth in GCBT was associated with higher odds for loss of all diagnoses.

**Table 4:** Loss of All Diagnoses at Long-Term Follow-Up Predicted by Predictor Variables

		*OR*	*p*	*OR 95% CI*	
Therapist-rated alliance with youth (TASCtc)		1.02	0.941	0.66	1.56
Youth-rated alliance with therapist (TASCct)		1.09	0.766	0.62	1.92
Adherence (Adh)		0.91	0.775	0.49	1.71
Competence (Comp) ^a^		0.77	0.388	0.42	1.40
Formal CBT training (CBT)		1.86	0.050	1.00	3.44
Clinician Severity Rating (CSR)		1.00	0.994	0.67	1.49
Group treatment (Group)		0.65	0.069	0.41	1.03
Adh*Group		0.48	0.058	0.22	1.03
Adh*Comp		1.41	0.112	0.92	2.16
Comp*CBT		0.92	0.861	0.37	2.28
Comp*Group		0.72	0.388	0.34	1.53
Comp*CSR		1.04	0.862	0.70	1.53
TASCtc*Group		2.75	0.001	1.52	4.95
TASCct*Group		1.38	0.492	0.55	3.44

*Note.* Loss of all diagnoses at long-term follow-up predicted by youth-rated alliance, therapist-rated alliance, adherence, competence (residualized), formal CBT training, clinician severity rating, and the effect of individual treatment compared to group treatment. Hypothesized interaction terms are included. Continuous predictors are standardized

^a^ Residualized variable after accounting for adherence

### Loss of Principal Diagnosis at Long-Term Follow-Up

Loss of principal diagnoses was not found to be significantly related to any main effect predictors (Table 5). Three interaction effects were found. First, adherence was found to be related to loss of principal diagnosis for GCBT, indicating that higher levels of adherence was associated with higher odds for loss of principal diagnosis in GCBT. Second, although adherence and competence were not related to loss of principal diagnosis as main effects, the adherence x competence interaction was. This interaction indicates higher odds for loss of principal diagnosis if both adherence and competence is high. Third, the results showed that therapist-rated alliance was related to positive outcome for GCBT, indicating increased odds for loss of principal diagnosis for higher reported levels in therapist-rated alliance with the youth for GCBT.

**Table 5:** Loss of Principal Diagnosis at Long-Term Follow-Up Predicted by Predictor Variables

		*OR*	*p*	*OR 95% CI*	
Therapist-rated alliance with youth (TASCtc)		0.96	0.842	0.61	1.49
Youth-rated alliance with therapist (TASCct)		1.25	0.504	0.65	2.43
Adherence (Adh)		0.87	0.689	0.43	1.75
Competence (Comp)^a^		0.91	0.789	0.46	1.81
Formal CBT training (CBT)		1.81	0.061	0.97	3.36
Clinician Severity Rating (CSR)		0.91	0.622	0.61	1.35
Group treatment (Group)		1.04	0.860	0.65	1.67
Adh*Group		0.43	0.030	0.20	0.92
Adh*Comp		1.85	0.016	1.12	3.05
Comp*CBT		1.14	0.747	0.52	2.53
Comp*Group		0.46	0.058	0.21	1.03
Comp*CSR		1.16	0.494	0.76	1.78
TASCtc*Group		2.17	0.012	1.18	3.97
TASCct*Group		1.28	0.617	0.49	3.37

*Note.* Loss of principal diagnosis at long-term follow-up predicted by youth-rated alliance, therapist-rated alliance, adherence, competence (residualized), formal CBT training, clinician severity rating, and the effect of individual treatment compared to group treatment. Hypothesized interaction terms are included. Continuous predictors are standardized

^a^ Residualized variable after accounting for adherence

## Discussion

The first aim of this study was to investigate whether therapist adherence, therapist competence, and therapist-youth alliance predicted outcome at long-term follow-up in CBT for anxiety disorders in youth. The results indicated that neither therapist adherence, therapist competence, nor the alliance predicted long-term follow-up when these variables were investigated separately. Given the mixed results across previous studies investigating adherence and competence as predictors of outcome, the research questions related to adherence and competence were explored openly. The present finding that neither of these variables predicted outcome is in line with several prior studies (e.g., Southam-Gerow et al., 2021). However, the finding that the alliance did not predict outcomes was contrary to our expectation. The null-findings may be due to the long-term perspective on our outcomes. The highest range of our follow-up measures was > 5 years post-treatment, and variables measured during treatment may be less relevant than variables that are closer in time, such as academic/work status, life events, and quality of life. However, some interesting findings concerning our second aim showed that the zero-findings may be due to lack of consideration of interaction effects.

The second aim was to examine if there were interaction effects between the predictor variables and treatment format (GCBT versus ICBT), baseline symptom severity and formal CBT training. None of these variables predicted long-term follow-up when considered alone. However, several interaction effects were found. Therapists displaying both high adherence and high competence achieved better long-term outcomes in terms of loss of principal diagnosis. Traditionally, research has investigated adherence and competence as separate predictors, and the results from the current study suggest that although none of these variables predict long-term outcomes when considered alone, the combination of displaying high adherence and high competence does. Fairburn & Cooper (2011) discussed that the distinction between adherence and competence may be less meaningful to everyday clinical practice if the overall standard of the treatment is of concern, and they favored abandoning this distinction and rather focus on “the extent to which a psychological treatment was delivered well enough for it to achieve its expected effects” (p. 374). They argued that high adherence is of little interest if delivered with low competence, and that doing the wrong things (i.e., being non-adherent) in a competent manner also is of limited clinical relevance (Fairburn & Cooper, 2011). The current results support this notion, indicating that it is the combination of doing the right things (adherence) with high competence, that is related to long-term CBT outcomes.

It could be argued that our findings regarding adherence and competence may only be relevant for clinicians using treatment manuals. A survey among U.S. mental health clinicians (*N* = 756) from different theoretical orientations found that less than 10% of therapists routinely used treatment manuals, although most clinicians reported using manuals to some degree (Becker et al., 2013). However, Becker et al., (2013) also found that only a CBT theoretical orientation was associated with being a frequent treatment manual user, and 63% of CBT therapists indicated being frequent users This may suggest that our findings are particularly relevant to CBT providers.

Regarding treatment format, the interaction effects suggested that predictors could play different roles for long-term follow-up outcomes following GCBT versus ICBT. Therapist-rated alliance was related to long-term outcomes for both loss of principal diagnosis and loss all diagnoses when treatment was given in groups. This suggests that alliance may be particularly important in GCBT for anxiety disorders in youth. Correlational results indicated that receiving GCBT was associated with youth-rated alliance. Thus, increasing alliance in GCBT could be a clinical implication that may increase the effectiveness of GCBT. It is particularly interesting that the alliance finding only applied to GCBT. On the one hand, the finding may appear counterintuitive. There is less room for individual therapist-patient relations in GCBT, and other processes such as group cohesion may be more relevant process variables in this format (Burlingame et al., 2018). On the other hand, precisely the fact that the patient “competes” with other patients in creating a bond with the therapist may help explain that better alliance predicted diagnostic recovery in GCBT only. Importantly, the finding only applied to therapist-rated alliance, and not to youth-reported alliance. This means that those patients the therapist felt the strongest bond with did better at long-term follow-up. It is also possible that the most engaged group participants (e.g., responding to questions and partaking in discussions) score higher on therapist-rated alliance by virtue of increased contact. Our findings suggest that exploring the role of the alliance for GCBT outcomes may represent a path towards identifying evidence-based process factors.

In ICBT, the results suggested that adherence was particularly important for loss of principal diagnosis, whereas it was not in GCBT. Although speculative, it may be that because ICBT gives more room to work individually tailored on the various CBT components (e.g., cognitive restructuring, exposure tasks), adherence plays a more important role in ICBT. Clinicians have been found to reduce adherence to treatment manuals over time (Chu et al., 2015) and the finding that adherence in ICBT was particularly important for loss of principal diagnosis should be noted as a clinical implication.

Therapists’ formal CBT training did not predict outcomes at long-term follow-up. This suggests that receiving treatment protocol training and supervision specific for treating youth anxiety disorders may be sufficient to provide effective long-term treatment. All study therapists had a relatively high level of training in the CBT protocol, which could indicate that reaching a certain level of CBT training is sufficient and resulting in formal CBT training not contributing to better long-term outcomes. Type of treatment format did not predict any outcomes at long-term follow-up, which is in line with post-treatment results in youth anxiety treatment (James et al., 2015). Furthermore, anxiety symptom severity at baseline did not predict long-term follow-up, which is contrary to post-treatment CBT outcomes in youth with anxiety and depressive disorders (Kunas et al., 2021).

We found that the interaction effects predicted loss of principal diagnosis but not reduced anxiety symptom ratings (child and parent). This finding seems counterintuitive, given that loss of diagnosis should also reflect symptom improvement. This finding may be an effect of how symptoms and diagnosis were assessed. Diagnosis was based on the ADIS-IV interview, while symptoms were assessed with the SCAS. Prior research has demonstrated that the convergent validity between SCAS-rated symptoms (child and parent) and clinician rated disorder (ADIS-IV) is significant and satisfactory (Brown-Jacobsen et al., 2011). However, the clinical utility of the SCAS-parent reported symptoms in predicting clinical diagnosis was estimated to approximately 60%, and even lower for child reported symptoms (Brown-Jacobsen et al., 2011). Thus, the noted finding may be an artefact of this semi partial overlap between SCAS and ADIS-IV.

Prior research on predictors of long-term outcome following CBT for youth anxiety is still limited, and results are mixed. Some predictors associated with outcome have been identified, although these results are in most cases found in single studies, as opposed to across studies. Predictors that have been identified in more than one study, include patient factors such as negative life events during follow-up period and a diagnosis of social anxiety, both predicting negative long-term outcome, while post-treatment remission has been found to be associated with a positive long-term outcome (Ginsburg et al., 2018; Kendall et al., 2004; Kodal et al., 2018b). Predictors of outcome, in the form of gender, age, severity of youth anxiety at pre-treatment, principal diagnosis, comorbidity, externalizing disorder/symptoms and family functioning, have demonstrated mixed findings (Barrett et al., 2001; Benjamin et al., 2013: Ginsburg et al., 2014; Ginsburg et al., 2018; Kendall & Southam-Gerow, 1996; Kendall et al., 2004). Future research could investigate whether there are interaction effects present in these predictors in order to enhance treatment effects through increased knowledge regarding “what works for whom?”. To date, studies on predictors of outcome in CBT for youth anxiety disorders have rarely looked at interaction effects. Such effects may be a key to advancing our understanding of how to improve treatments (Banneyer et al., 2018).

The current study has several strengths, including the use of an RCT design with a large clinical sample and assessment methods with well-established psychometric properties. Four measurement points were included in the models to make the model estimations more precise. Furthermore, the longitudinal design with a long-term follow-up assessment gave us the opportunity to examine long-term predictor effects, which has not been subject to much previous research. The current study also included alliance as a predictor, which has been suggested to reduce the effect of adherence and competence on outcome (Webb et al., 2010).

There are also limitations. These include the therapists being White and mainly female, possibly limiting generalizability of the results. Furthermore, the sample consisted of limited diversity (90.7% White) and participants were from primarily medium-high SES. The results are also limited to the anxiety disorders treated in the current study. Further research is warranted to investigate the role of the included predictors for other anxiety disorders. Furthermore, as adherence and competence were rated by the same rater due to limited resources, instead of applying different raters to measure the two variables, this may possibly influence the correlation between adherence and competence. The raters also assessed the same patient and the same therapist more than once, indicating that the sample of rated sessions is not independent, which can introduce a possible rater confound (Consbruch et al., 2012). As the concurrent validity of the CAS-CBT has not been investigated, other scales for measuring adherence (Southam-Gerow et al., 2016) and competence (McLeod et al., 2016) for youth anxiety could be used to assess concurrent validity of the CAS-CBT in future studies. It should also be noted that the research literature has questioned whether alliance predicts outcome, or whether positive outcomes may predict higher alliance (Zilcha-Mano et al., 2014). As the current study was not designed to include multiple assessment points of both alliance and outcome throughout the treatment, we were unable to investigate the direction of effects. Further, as the treatment involves several parent-components (e.g., parent-sessions and parents’ involvement in homework tasks) it would be useful for future studies to examine if parent-reported alliance is associated with outcome. Lack of statistical power may also represent a limitation. A priori Monte Carlo power analysis was not conducted as no earlier comparable studies with main- and interaction effects could be used as population parameters. We did not correct for multiple testing, which would have increased the type II error rate. On the other hand, we did not remove non-statistical interaction terms, which could “hide” statistically significant main effects. This study is exploratory and later studies should therefore be conducted to confirm or disconfirm the presented findings.

In the present study, we found that therapists displaying both high levels of adherence and competence achieved better long-term outcomes, and that alliance between therapist and youth may be particularly important for long-term outcomes in GCBT, whereas adherence may be particularly important for long-term outcomes in ICBT. These results indicate that interaction effects should be subject to more research in this area. The study has several clinical implications for CBT for youth anxiety disorders. Therapists should be supported through training and supervision to enhance both adherence and competence, as they may achieve better long-term outcomes this way. In GCBT, therapist behaviors that facilitate alliance (e.g., Fjermestad et al., 2021) may be particularly important to enhance in treatment. In ICBT, therapist behaviours that could facilitate high adherence to treatment may need more focus. Service providers should consider targeting these factors when implementing CBT for youth anxiety disorders.

## Electronic Supplementary Material

Below is the link to the electronic supplementary material.

Supplementary Material 1
