# Supplementary material for: Adherence, Competence, and Alliance as Predictors of Long-term Outcomes of Cognitive Behavioral Therapy for Youth Anxiety Disorders
Source: Res Child Adolesc Psychopathol. 2023 Jan 24;51(6):761–73. doi: 10.1007/s10802-023-01028-1 (PMC10195754; doi:10.1007/s10802-023-01028-1)
Supplement: Supplementary file 1 — Supplementary Material 1 [file 10802_2023_1028_MOESM1_ESM.docx]

# Supplementary material

**Table S1.**

*Parent-Reported Anxiety Symptoms (SCAS) from Pre-Treatment to Long-Term Follow-Up Predicted by Predictor Variables*

|  |  | *b* | *p* | *95% CI* | |
| --- | --- | --- | --- | --- | --- |
| Intercept |  | -0.19 | .023 | -0.35 | -0.03 |
| Therapist-rated alliance with youth (TASCtc) |  | 0.02 | .282 | -0.02 | 0.06 |
| Youth-rated alliance with therapist (TASCct) |  | -0.02 | .332 | -0.06 | 0.02 |
| Adherence (Adh) |  | -0.23 | .564 | -1.00 | 0.54 |
| Competence (Comp) ^a^ |  | 0.16 | .427 | -0.23 | 0.54 |
| Formal CBT training (CBT) |  | -0.02 | .707 | -0.13 | 0.09 |
| Clinician Severity Rating (CSR) |  | -0.01 | .476 | -0.03 | 0.01 |
| Group treatment (Group) |  | -0.01 | .828 | -0.10 | 0.08 |
| Adh*Group |  | 0.09 | .719 | -0.41 | 0.60 |
| Adh*Comp |  | -0.02 | .474 | -0.07 | 0.03 |
| Comp*CBT |  | -0.09 | .050 | -0.17 | 0.00 |
| Comp*Group |  | -0.05 | .747 | -0.32 | 0.23 |
| Comp*CSR |  | 0.01 | .211 | -0.00 | 0.01 |
| TASCtc*Group |  | -0.02 | .142 | -0.04 | 0.01 |
| TASCct*Group |  | 0.00 | .731 | -0.02 | 0.03 |

*Note.* Parent-reported anxiety symptoms (SCAS) from pre-treatment to long-term follow-up predicted by youth-rated alliance, therapist-rated alliance, adherence, competence (residualized), formal CBT training, clinician severity rating, and the effect of individual treatment compared to group treatment. Hypothesized interaction terms are included.

^a^ Residualized variable after accounting for adherence.
